# Optimization on Personal Fall Arrest Systems. Experimental Dynamic Studies on Lanyard Prototypes

**DOI:** 10.3390/ijerph17031107

**Published:** 2020-02-10

**Authors:** Juan Carlos Pomares, Elena Ángela Carrión, Antonio González, Pedro Ignacio Saez

**Affiliations:** 1 Civil Engineering Department, University of Alicante, P.O. Box 99 E-03080 Alicante, Spain; antonio.gonzalez@ua.es (A.G.); pi.saez@ua.es (P.I.S.); 2 Building & Urban Development Department, University of Alicante, P.O. Box 99, E-03080 Alicante, Spain; elena.carrion@ua.es

**Keywords:** personal protective equipment, fall arrest systems, dynamic performance test, lanyard, low stretch kernmantle and dynamic rope, webbing

## Abstract

Tens of thousands of fall-from-height accidents take place at construction sites every year. These types of accidents range from minor to fatal, causing a significant financial burden to enterprises, personal and family traumatic experiences, high medical costs, as well as hard compensation claim settlements. It makes sense then, that some sort of effective personal protective equipment (PPE) be devised to stop these types of accidents from happening. This article aims to explain how PPE can be used to minimize personal injury and the costs implied. The main contribution of this study is that the prototypes made with dynamic ropes and terminals knotted—without an energy absorber—could safely retain falls. Results show that standards EN 354 and EN 364 need to incorporate dynamic test requirements, for the reason that a high loading rate significantly reduces the resistance in static tests that manufacturing companies claim they have. Surprisingly, more than 90 percent of work at heights use PPE without any absorber. Finally, this study calls for the need to accurately determine the dynamic response of PPE in order to further advance in improvements of these fall arrest systems with no energy absorber.

## 1. Introduction

Falls from a height are the main causes of serious and fatal workplace accidents in almost every country. In the USA [1], fatal workplace injuries from falls, slips and trips have continued a general upward trend, with an increase of 6 percent, and an overall increment of 25 percent in the last 10 years. A study of occupational activity fatalities per year in different countries describes quite a similar scenario. Generally speaking, the year 2016 showed an increase in fatalities of over 25 percent in production sectors, such as roofers, carpenters, tree trimmers and others. In the United Kingdom [2], the statistics for the construction sector show that falls from a height have represented roughly 50 percent of the total fatal accidents.

Taking the construction sector in the USA as a reference, accidents due to falls from heights are the cause of the greatest number of deaths for the years 1990 and 2001. These accidents seem to correlate with the number of workers in the sector [3]. This trend persisted in the period between 1997 and 2012, where accidents due to falls from heights went from 36.3 percent in the Huang study [3] to 44.6 [4]. In Australia [5], the fall-from-height fatality rate was 14 percent in 2016, and was only surpassed by motor collision casualties. In Singapore [6], falls from heights have been the greatest cause of fatal injuries—31.9 percent—in the last ten years. In Spain [7], statistics for the year 2017 reveal that construction sector accidents represent 17.66 percent of all serious accidents, of which 10.64 percent end in the workers’ deaths. Specifically, to reduce the number of accidents due to falls from heights, the correct use of fall protection equipment is indispensable [8,9,10]. Currently, the use of personal protective equipment (PPE) against falls remains a pending subject. Analysis of near-accidents and dangerous behaviors shows the need to simplify this equipment, if the correct use of it by workers is to be increased [11,12]. For this equipment to be used correctly, it must be not only simple but also easy to use [9]. 

Choi et al. [13] published a detailed, comparative study on accidents from 2011 to 2015 in the United States, Korea and China. Results showed that the construction industry had consistently high fatal occupational injuries, and the top most common accident types were “fall from a higher level” and “struck by”. The typical temporary work in the construction sector has a negative impact on the injury frequency index, so much so that the index for temporary workers increases between 136.4% and 175.2% [14] more than the value found for direct employees in the most hazardous industrial sector.

Previous collective protection equipment studies, such as the one carried out by [15] that complements PPE is also considered, even though its approach is presented from a different perspective. In this general context, PPE against falls from a height, in particular fall arrest systems, have become a must to control the safety of workers [16,17] where each of the components in the system is critical to protect the worker. The simplest system consists of (a) a full-body harness, (b) connectors, (c) lanyards, (d) a shock absorber, and (e) an anchor [18,19]. Different papers have specifically focused on each of the components. For example, shock absorbers [19,20], harnesses [21,22], connectors [23], anchor devices [24] and even a multi-component system [17,25]. 

Destructive experiments frequently use small sample sizes due to the cost of test specimens and the time and resources involved. Goh and Peter [19] evaluated seven types of energy absorbers. Baszczynski [26] studied four types, using two or three samples for each simulated condition. Riches [27] used only one fall arrest sample in seven different configurations. Authors like Baszczynski [28] tackled adjustable lanyards of low elongation. 

In the tests performed for this paper, fall factor (FF) has been considered as the ratio between the height of fall H, before the equipment is put into tension, and the length L of the equipment that absorbs the fall energy: FF = H/L. This criterion is used by American National Standards Institute (ANSI) A10.32:2004 [29] and it is perfectly adapted to the needs of the studies carried out, and it also allows for a correct exposure of the results. Therefore, in a lanyard where the length is constant, the FF will oscillate between the values 0 and 2.

**Figure 1:**
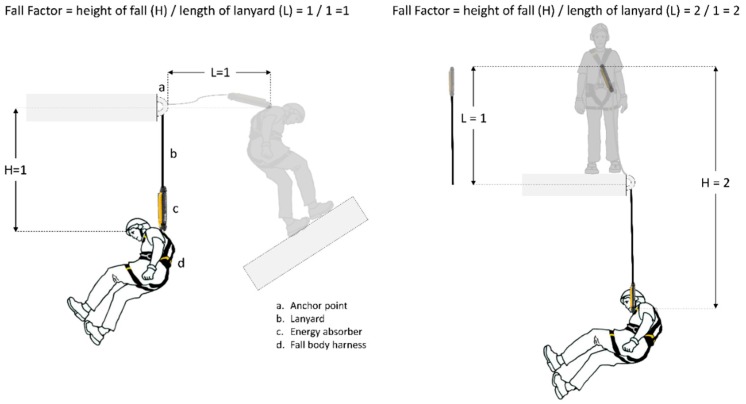
Fall factor (FF) studied. FF = 1 (**left**) and FF = 2 (**right**).

In Figure 1 different FFs have been represented with fall arrest equipment with a length of 1 m.

The main function of a fall arrest system is to safely arrest the fall. To achieve this purpose, different standards are set to limit the force of impact. These limits represent the assumed maximum impact that a person can withstand without undergoing much harm. Table 1 shows the maximum admissible forces for each standard.

Table 2 shows standards in terms of static resistance of lanyards, which must necessarily be equal to or greater than 22 kN. 

Other safety problems in work at heights, detected by several authors, include unawareness on how to use energy absorbers properly, thus making the system unsafe. Studies conducted by the Spanish National Institute of Occupational Health and Safety by Jiménez et al. [38] show that of the 1117 construction sites visited, 17 percent made proper use of the fall arrest system. Shockingly, they also found that 81% placed the anchor point incorrectly; 21% made use of an anchor line; 13% made use of a sliding fall arrest system, which was inadequate, and 6.7% incorporated an energy absorber.

Surprisingly, more than 90 percent of work at heights [38], with risk of falling, use PPE without any absorber. This study aims to determine what a lanyard of a low stretch kernmantle and dynamic ropes needs to absorb the required energy to arrest a fall safely. This article focuses on the design of the lanyards with ropes from the world of sport (with a higher energy absorption capacity) as a replacement of energy absorbers. The main point of this design is to improve the safety of PPE, without any absorber, used during a hypothetical fall of a worker.

## 2. Materials and Methods

### 2.1. Lanyards Studied

**Figure 2:**
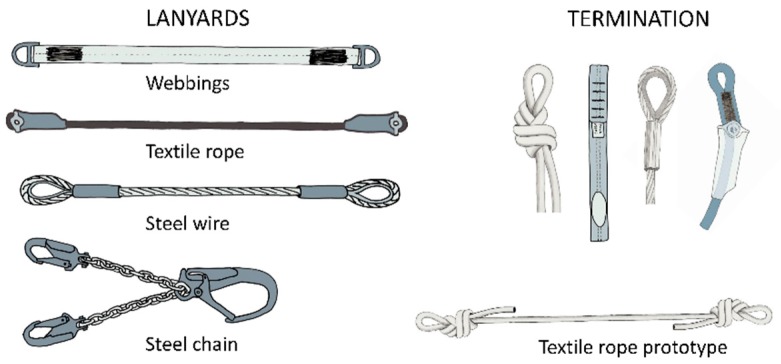
Different lanyards and terminations commonly used.

In terms of materials used and terminations, lanyards can be of four types (Figure 2): Webbings, commercialized and prototype textile rope, steel wire and steel chain. Webbings are mainly made of polyamide (PA) and polyester (PTA). Their terminations are made in the form of a gauze where connectors fasten the anchor point and the harness.

The dimensions of the lanyards under consideration are: Diameter of the ropes between 9–12 mm, webbing width between 15–40 mm, and lengths between 0.50–1.8 m, leaving connectors out. The main characteristics of the low stretch kernmantle and dynamic ropes stated by EN 1891 [39] and EN 892 [40] are shown in Table 3.

Ropes used for canyoneering, caving or rope access and positioning, with low stretch kernmantle behavior, are regulated by EN 1891 [39]. Ropes used for mountaineering and climbing, with dynamic behavior, are regulated by EN 892 [40] in Europe. Both types of ropes are made of natural raw fibers which are marketed without manufactured terminals. It is the user who makes a knot as a terminal. The most common and recommended knots are type “eight” for its simplicity of construction and ease of undoing when the lanyards are subjected to loads. This type of eight knot when used as an anchor knot shows a residual static resistance (Ro) in the range of 62–67% of nominal rope resistance without knots (with a diameter of 10.5 mm) [41,42]. This study aims to determine if it is possible to make lanyards of a low stretch kernmantle or dynamic rope that absorb the required energy to arrest a fall safely.

Sixteen specimens were produced for testing, eight for FF = 1 and eight for FF = 2. Two rope prototypes 1 (PT1) in compliance with the European Standard EN 1891 A [39], braided ropes with sheath, low stretch kernmantle; and two rope prototypes 2 (PT2) in conformity with the European Standard EN 892 [40], dynamic ropes, safety requirements and test methods, with knotted terminals. Both, PT1 and PT2, were designed by the authors of this paper. With regard to the certificate lanyards selected by the authors, they are of two types: T1 rope, complying with the European Standard EN 354 [36], and two T2 webbing, in conformity with EN 354 [36]. Both, T1 and T2, were selected from manufacturing companies.

The connectors used in the tests meet the standard EN 362 [43] requirements, which describe technical specifications for connector requirements, test apparatus, test methods, marking, and manufacturing-companies-supplied information for connectors (static strength of major axis is 25 kN).

Unused test samples have been engaged in every dynamic test. The data was recorded automatically through a force measuring apparatus at every 2 ms.

All tested lanyards and prototypes were checked 24 h in temperature and humidity-controlled chambers, in accordance with EN 364 [44]. Table 4 shows the main specimen specifications.

**Figure 3:**
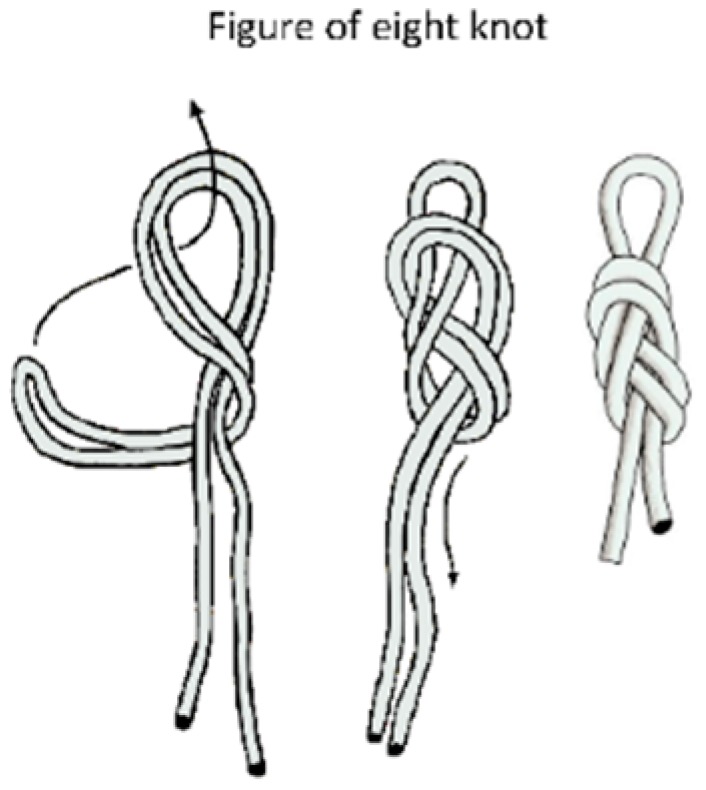
Knot commonly used as a terminal: Type eight.

Figure 3 illustrates a figure-of-eight knot terminal at both ends of the prototypes, featured with rope following recommendations of several authors [42,43,45] since this type of knot is not only easy to make but also easy to check.

**Figure 4:**
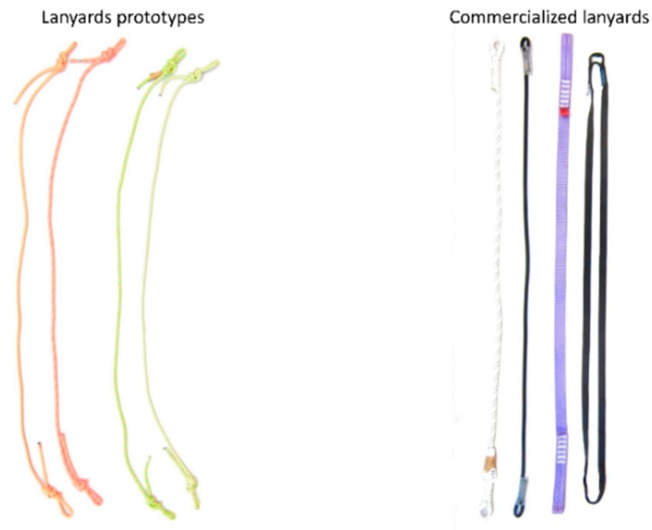
Prototypes (**left**) and commercialized lanyards (**right**).

Figure 4 shows both the prototypes and the commercialized lanyards being tested. The knots in the lanyard prototypes (PT1 and PT2) have been made by experts, ensuring homogeneity.

Lanyards, ropes, and connectors fall into Category III in the European Regulation 2016/425 231 [46]. This code establishes that small sample sizes are acceptable. The regulation also states that a certification agency either carry out product checks to verify production and personal protective equipment (PPE) homogeneity, or assess the manufacturing companies claim in terms of system quality to determine whether the requirements are satisfied.

### 2.2. Dynamic Testing Apparatus Used

**Figure 5:**
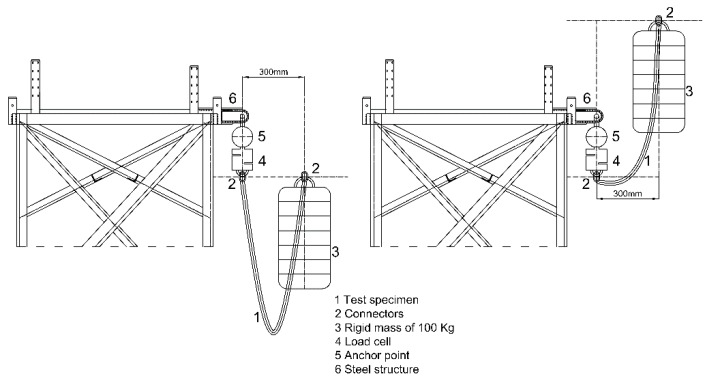
Test procedure FF = 1 (**left**) and FF = 2 (**right**).

The authors designed the rigid structure of the apparatus (Figure 5), which complies with the standard EN 364 [44], which means that the application of a 50 kN load on the anchorage causes a vertical deflection of less than 1 mm, and a natural frequency (of vibration) in the vertical axis of 100 Hz at the anchorage.

The anchor point, equipped with a spherical ball working like a hinge, allows free oscillation, as indicated in EN 364 [44] and EN 355 protocols [34].

The test mass having a diameter of 200 mm is cylindrical in shape and is made of steel. The weight simulates that of a person falling. The selected design allows different configurations for mass to be progressively added (in the range of 50 to 150 kg) when required.

The load cell is made by HBM (Hottinger Baldwin Messtechnik) [47] type RSCC and it resists a maximum force of 50 kN. The control and data analysis software used is PCD2K (Servosis company, Madrid, Spain) from Servosis Testing Machines [48]. This software has a control frequency of up to 40 kHz.

### 2.3. Dynamic Performance Test

The test method chosen for the experiments correlates with the one described in EN 355 [34] and EN 364 [44], enabling a comparison of the resulting impacts in different test samples. Commercialized lanyards of equal lengths, 1500 mm, have been selected. Table 5 shows the lengths (without connectors)—as specified by the manufacturing companies—and a rigid mass of 100 kg.

Test Procedure: A 50 kN load cell was connected to an anchor point by means of a spherical ball and a socket joint. One end of the lanyard terminal was connected to the load cell and the other end to the mass (Figure 5).

The mass connected to a quick release system was suspended from a lanyard to be raised to a certain height to study both FF = 1 and FF = 2 impacts, to establish similarities and contrasts between the two scenarios. Figure 5 shows the arrangements schematically. The mass was placed 300 mm away from the anchor point in the horizontal plane. When the mass was dropped, it was checked whether the mass was successfully arrested by the system and the values for maximum arrest force, together with the behavior curve force/time, could readily be calculated. In addition, post-test elongation for each lanyard was measured.

Lanyard post-test elongation values were obtained by calculating the difference between the initial length and the length at rest after completion of the test. Table 5 illustrates the initial length measurements of the sample suspended from the test gantry, subjected to its own weight. In this way, the regulations in-tension measurement requirements in the absence of a load were fulfilled. This measurement includes the connectors from the end points that withstand the load.

Post-test length measurements are performed in the same manner when the mass has stopped. Table 5 and Table 6 show values of the lanyards elongations with a calibrated flexometer in millimeters, connectors included.

In summary, different samples (PT1, PT2, T1 and T2) have been tested with the objective of checking whether a fall is successfully arrested, so as to minimize the risk currently run by workers involved in tasks done at heights, where a fall from the line of work in rope access systems may mean serious injury. The tests inform whether fall arrest systems can be simplified for cases where energy absorbers may be redundant.

## 3. Results 

### 3.1. Fall Factor 1 Tests (FF = 1)

In every test in FF = 1, the mass was retained. Table 5 illustrates the maximum arrest force and elongation obtained for lanyard commercialized and textile ropes prototypes. 

Tests 1 and 2 (PT1) reach 8 and 8.6 kN in peak of arrest forces. Tests 3 and 4 (PT2) exhibit maximum arrest force values of 6 and 7 kN. Their equipment shows an elongation higher than 20 percent. Considering the limit of 8 kN, these prototypes could retain the load stretching the lanyard up to 1.5 m in FF = 1.

Prototypes PT1, made with low stretch kernmantle ropes show that their elongation is reduced by 5 percent with respect to PT2, increasing, at the same time, the maximum arrest force by 14 percent at 8 kN, approaching the limit allowed by the ANSI Z359.1 [30] and ANSI Z359.13 standards [31]. Table 5 shows the maximum arrest force and elongation for all specimens in FF = 1.

**Figure 6:**
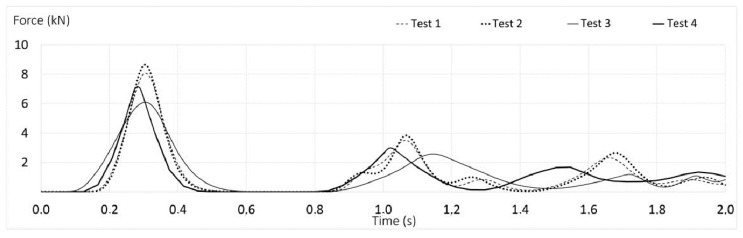
Time-force curves of Tests 1 to 4 (textile lanyards prototypes) with FF = 1.

Figure 6 shows the force-time curves for the prototypes studied. Test 2 (PT1) shows a maximum force around 8 kN and Tests 1, 3 and 4 present forces equal to or lower than 8 kN. Tests 3 and 4 (PT2) present a smoother curve and, therefore, a lower maximum arrest force. At first impact, the time lapse is 0.3 s, and then the maximum retention force is obtained. The second and successive rebounds present considerably lower forces.

**Figure 7:**
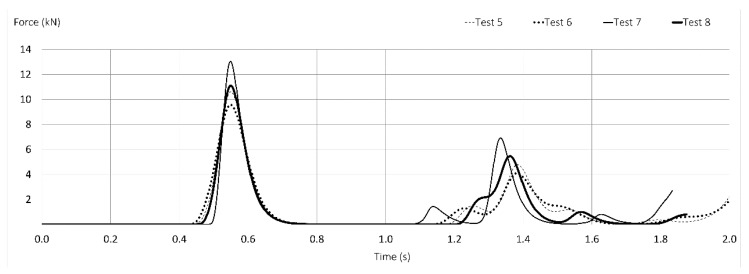
Time-force curves of Tests 5 to 8 (commercialized lanyards) with FF = 1.

As can be appreciated in Figure 7, the commercialized lanyards present curves with larger slopes. Test 7 (T1) and Test 8 (T2), commercialized with webbings, present the highest maximum arrest forces—13 and 11 kN—with practically no elongation (less than 2.5 percent). Test 7 (T1) exceeds 8 kN widely at first impact and again exceeds 6 kN in the second rebound.

Less than 1 s after the first impact, in both figures (Figure 6 and Figure 7), a second important rebound is observed, in which the value of the force generated is approximately half the force of the first impact.

All the commercialized lanyards (T1) based on ropes, Tests 5 and 6, show small elongation, less than 8 percent, behaving better than webbings (Tests 7 and 8), but the values obtained in textile rope prototypes (PT1 and PT2) are greater than 15 percent. Commercialized lanyards (T1 and T2) by themselves are unable to maintain the maximum arrest force at tolerable values.

### 3.2. Fall Factor 2 Tests (FF = 2)

Not all lanyard tests evaluating FF = 2 were able to retain the mass successfully, i.e., without snapping. Table 6 shows the maximum arrest forces or snapping forces and the elongations taking place in the samples after the tests had been completed.

Restricting this study to only those having a length of about 1.5 m, prototypes (PT1 and PT2) group (Tests 9, 10, 11, 12), the results are better than those obtained in the commercialized (T1 and T2) group (Tests 13, 14, 15, 16), for the reason that in the first group, only one prototype snapped, while in the second group, three commercialized lanyards failed. In addition, among those that retained the load, the maximum arrest force presented low values in the first group.

Test 11 (PT2) and Test 12 (PT2) showed the best responses, as was the case when FF = 1 was analyzed. FF = 2 for Test 11 showed a strain of 0.38 and a maximum force of 8521 N almost succeeds in retaining a load falling from over 3.5 m in freefall.

**Figure 8:**
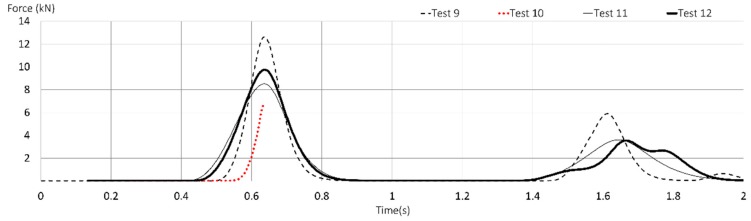
Time-force curves of Tests 9 to 12 (textile lanyards prototypes) with FF = 2.

Figure 8 refers to the dynamic behavior of prototypes subjected to FF = 2. As can be seen, the mass was retained except for Test 10 (PT1 made with low stretch kernmantle rope). Test 9 (PT1) showed a maximum arrest force of 12,632 N with an elongation of 20.87 percent. However, none of these four lanyards (PT1 and PT2) were able to retain the mass at tolerable forces, lower than 8 kN.

**Figure 9:**
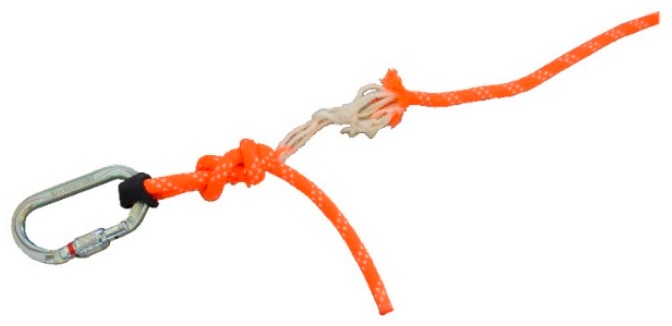
Failure of specimen in Test 10.

In Test 10 (PT1), the failure occurred in the strangulation of the first knot (Figure 9), under 7000 N, which means a reduction of more than 75 percent in static resistance, contrary to what the manufacturing companies claim. Furthermore, the core of the ropes underwent damage in the contact area of the connector.

**Figure 10:**
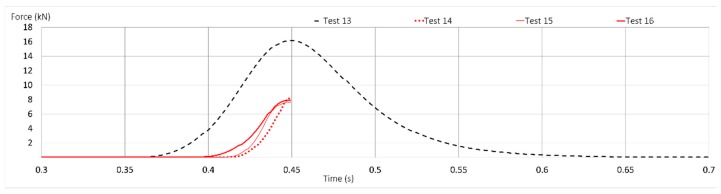
Time-force curves of Tests 13 to 16 (commercialized lanyards) with FF = 2.

Figure 10 illustrates the results of the forces reached in commercialized lanyards (T1 and T2) under FF = 2. In this figure, a shorter period of time span helps visualize the different time-force curves with greater clarity.

**Figure 11:**
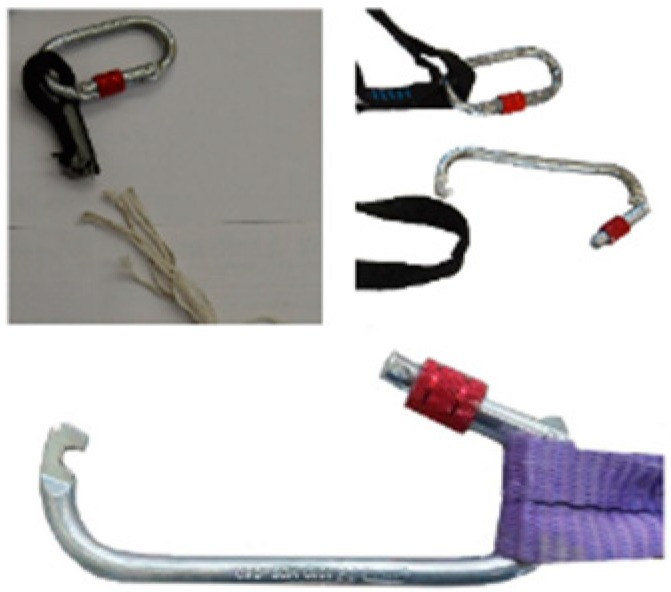
Lanyards connectors of post-test 14 (**top left**), 15 (**bottom**) and 16 (**top right**), respectively.

The results of lanyards of length around 1500 mm are shown, Test 13 (T1) shows that the rigid mass was retained at a maximum arrest force of 16,178 N and a strain of 0.06 (Table 6). This force observed is 4 kN higher than that for Test 9 using low stretch kernmantle rope (PT1). Moreover, Figure 10 also shows the samples after Tests 14, 15 and 16 were carried out. Test 14 (T1) failed at 8228 N. Test 15 (T1) failed at 5455 N in one of the two connectors (see Figure 11), which means a reduction of 79 percent resistance with respect to the static resistance compared with the connector values offered by manufacturing companies. In Test 16 (T2), the damage occurred at 7941 N, showing a reduction in static resistance of more than 68 percent of that declared by the manufacturing companies, and stipulated for connectors in static tests. The reason for these high values resides in the very stiffness of the webbings and the insufficient elongation the experiment required.

**Figure 12:**
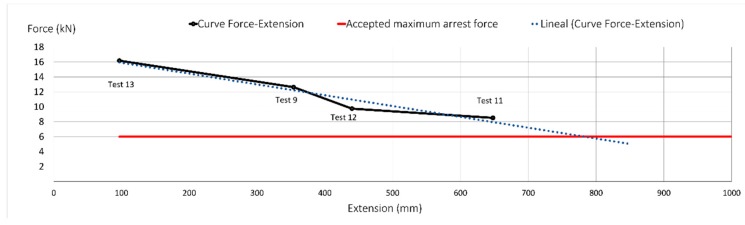
Force-elongation post-test (FF = 2).

As seen in Figure 12, according to Tests 9, 11, 12 and 13, the greater the elongation of the rope, the less the maximum arrest force is. None of the lanyards tested could arrest the mass at forces lower than 6 kN. According to the PPE extensions drawn in the Figure 12, an elongation of 800 mm is necessary; this is a similar value to the deflection studied by the authors for edge protection systems in construction work [15].

## 4. Discussion

It is a fact that lanyards by themselves do not arrest falls from a height, but in conjunction with energy absorbers they are suitable for use in fall arrest systems. Therefore, the dynamic behavior of lanyards must be taken into consideration. In any case, when a fall arrest system is used, an energy absorber must be included to reduce the maximum arrest force to values under those established—6 kN in Europe, and 8 kN in the United States of America. Moreover, the energy absorber must be used in any work in height at every instance to counteract poor usage, change of system or carelessness on the part of the worker. 

This study responds to the need to underline that the implementation of EN 354 [36] does not compel the system to feature fall shock absorption, let alone a lanyard dynamic requirement. However, Carrión [49] observed that a great number of rope access works may well be subjected to FF = 1 and even to FF = 2 in the worst scenario [45]. Even some specific and ordinary work-at-height may be subjected to FF of 0.3 and 1. In such cases, accidents are hardly avoidable.

It is evident from the tests carried out that the hand-made prototypes devised by the authors (textile rope lanyards) show a better dynamic behavior than commercialized lanyards, absorbing more energy, obtaining lower impact force values and higher elongations.

Elongation, energy absorption and maximum arrest force are closely linked, so that with greater elongation, more energy is absorbed, and less maximum arrest force is transmitted to the operator in the phenomenon of fall arrest. In order to reduce the arrest force, larger elongations are necessary, however, not so large that they make the use of the equipment inoperative. Maximum distance from anchorage to the ground is 6 m with 6 kN of maximum arrest force. To absorb more energy and reduce the stopping forces considerably, that distance would be increased so that the equipment would not be operational because it needs too much distance below the anchorage. 

Durability of the prototypes it is determined by the manufacturer of the ropes used to make the prototypes, and like any textile element, it depends on the hours of use and exposure to atmospheric agents.

Table 7 shows the average results of lanyards tested in this paper. Data have been ordered according to the following items: Type of lanyard and fall factor. Maximum arrest force, elongation and strain, in their average values from Table 5 and Table 6, have also been included in this table for a deeper study of the results.

The results of this research attempt to improve the behavior of PPE against falls from height. The prototypes studied are manufactured according to the same standards (EN 1891 or EN 892); hence results obtained have a low dispersion.

## 5. Conclusions

Prototypes of dynamic ropes and knotted terminals (with no energy absorber) could safely retain FF = 1 falls. The lanyard prototypes studied in this paper can solve certain work situations, in which human errors end up compromising worker safety. Errors of this type account more than 90 percent of the total cases [38].

Dynamic ropes, showing lower retention forces when FF = 2, achieve substantial good results. The prototypes of Tests 11 and 12 (PT2) with a length of 1.5 m (without connectors) show the best dynamic performance. These prototypes comply with the ANSI Z359.13 [31] standard requirements in that they do not transmit the maximum arrest force of 8 kN in FF = 1, in 1.5 m free fall, which entails an average reduction of over 19 percent in the maximum established.

Test 11 (PT2) with dynamic rope EN 892 [40] and figure-of-eight knots in FF = 2 showed slight damage at 8521 N of impact force, making it very satisfactory.

Certified lanyards made from webbings show a maximum arrest force about two times greater than that of prototype dynamic rope lanyards devised by the authors (83.2 percent higher) when FF = 1 under the standard EN 354 [36].

All specimens were rendered useless after completion of the tests. The worst result was at Test 13 (T1) under EN 354 [36], which retained the mass at a maximum peak force of 16,178 N, which implies possible serious or even fatal accidents in FF = 2 contexts. This doubles the impact force obtained in Test 11 (PT2).

Having considered all the lanyards, webbings (T2) have shown a poor dynamic behavior. Consequently, the authors discourage the use of webbings without energy absorbers for work at heights.

With regards to those tests in which the mass was successfully retained, the average maximum arrest force for FF = 2 was 32.3 percent higher than for FF = 1.

It is emphasized that the requirement of static resistance does not guarantee the mass to be retained. The commercialized lanyards, with a static resistance of 22 kN, can snap at much lower forces in dynamic tests, at 5.4 kN (75 percent lower). European EN and ISO standards should include dynamic requirements for all lanyards, not only for adjustable ones, as stated in ANSI standards. In view of the results obtained and assuming possible operator failures, contemplated in Europe standards, a further step can be taken to demand a dynamic requirement below 8 kN for FF = 1. The ANSI Z359.3 [35], ISO 10333-2 [33], EN 358 [37] and EN 354 [36] static resistance requirement of 22 kN is of little relevance as the resistance obtained in the dynamic tests performed is much lower.

Furthermore, this paper shows that the loading rate significantly reduces the static stress of some of the fall arrest components: a) EN 362 [43] connectors that fail at 28 kN in static tests, fail at about 5.4 kN in dynamic tests; b) EN 354 [36] lanyards that fail at 22 kN in static tests, fail at 8.2 kN in dynamic tests; and EN 1891 [39] low stretch kernmantle ropes that fail at 27.3 kN in static tests, fail at approximately 6.5 kN in dynamic tests.

Looking at the summary data in Table 7, with FF = 1 all tests retained the mass, while for FF = 2 only 50% of the tests were retained.

For the tests of FF = 1, the average retention force of the prototypes (PT) was 7.48 kN compared to the commercialized lanyards (T) which obtained an average force of 11.09 kN, this means an increase of 48.26%. For FF = 1 the prototypes (PT) have an average elongation of 314 mm and a strain of 18.3%. For this same FF, the commercialized lanyards (T) obtained a strain of 4%, which means a stiffness 4.57 times greater.

In the tests with FF = 2, for prototype lanyards (PT), the mass was retained in 75% of the tests. The average force and strain were 10.30 kN and 28%, respectively. At the commercialized lanyards (T), the mass was retained for only 25% of the tests. A force of 16.18 kN and a strain of 6% were obtained, which means a stiffness of 4.67 times greater.

The standards that oversee the situation depicted above should include a requirement of dynamic strength to address the forces generated when the loading rate is very fast. In particular, in standard EN 362 [43] for connectors, and in EN 354 [36] for lanyards, it should be included in the before requirement. 

According to the present study, the following minimum dynamic requirements are recommended: A 100 Kg mass in a FF = 2 falling from a height of 4 m should be retained, and neither the lanyard nor the connector should fail at maximum arrest peak force lower than 8 kN. 

The knot, when tightened during the fall, constitutes an energy absorption element to be taken into account in lanyards. However, knots as energy absorption elements, and connector failure have not been thoroughly studied and will be tackled in the future.

The conclusions presented in this document are a significant step towards improving the behavior of personal fall arrest systems. Among prototypes made with ropes with the same certification (EN 1891 or EN 892), no major differences in behavior have been appreciated, unsurprisingly due to the exhaustive controls on the production of Category III’s personal protection equipment (PPE). One of the requirements that guarantees the homogeneity of the production is the control of the PPE by a certification company.

In future works, it should be noted that in order to achieve conclusions with a larger scope, it would be advisable to extend the sample tested, increasing the number of manufacturers, different rope diameters and other countries’ standards.

## Figures and Tables

**Table 1:** Maximum arrest force according to different standards.

Standard	Type	Free Fall (H) (m)	Fall Factor	Maximum Arrest Force (kN)
ANSI A10.32:2004 [29]	NO	3.60	2	6.2
ANSI Z359.1:2007 [30]	NO	1.80	1	8.0
ANSI Z359.13 2009 [31]	6 ft. free fall	1.80	1	8.0
	12 ft. free fall	3.60	2	8.0
Z259.11–17 [32]	-	variable	-	8.0
ISO 10333–2:2000 [33]	Type 1	1.80	1	4.0
	Type 2	4.00	2	6.0
EN 355:2002 [34]	-	4.00	2	6.0

**Table 2:** Static breaking strength of lanyards according to different standards.

Standard	Static Breaking Strength (kN)	Dynamic Strength			
		Test Mass (kg)	Fall Factor	Free Fall (m)	Requirement
ANSI A10.32 2004	-	100	1	-	Does not break and retain the mass
ANSI Z359.3 2007 [35]	22.2	136	-	1.22	Does not break and retain the mass
Z259.11–17	-	100	1	-	Arrest the fall of test mass without breaking
ISO 10333–2:2000	22	100	2	-	No tearing or rupture of lanyard,
					only if the lanyard is adjustable in length
EN 354:2011 [36]	22	100	2	4	Mass retained,
					only if the lanyard is adjustable in length
EN 358:2000 [37]	22	100	1	-	Mass retained

**Table 3:** Requirements of low stretch kernmantle and dynamic ropes.

Requirements EN 1891 for Low Stretch Kernmantle Ropes		Requirements EN 892 for Dynamic Ropes	
Diameter	8.5–16 mm	Percentage of core	≥50 %
Elongation 50–150 kg	≤5%	Elongation 80 kg	≤8%
Impact force FF = 0.3 (100 kg)	<6 kN	Impact force	≤12 kN
Number of falls FF = 1 (100 kg)	≥5	Number of falls FF = 2 (80 kg)	≥5
Static strength	≥22 kN type A (work ropes)	-	-
	≥18 kN type B (sports rope)		

**Table 4:** Test specimen specifications.

Specimen	Type	Static Strength (kN)	Rope Diameter (mm)	Webbing Width (mm)	Length (mm)	Material	Certification
1	PT1	27.3	9.9	-	1500	PA	EN 1891 A
2		28	10	-		PA-PTA	
3	PT2	-	10.1	-	1500	PA	EN 892
4		-	10	-			
5	T1	22	12	-	1500	PA	EN 354
6			11			PA-PTA	
7	T2	22	-	35	1500	PA	EN 354
8		25	-	25		PA-PTA	

**Table 5:** Test of specimens. Maximum arrest force and elongation in FF = 1.

Specimen	Type	Test	Length with Connectors (m)	Maximum Arrest Force (N)	Lanyard Elongation (mm)	Strain (m/m)	Terminal Type	FF	Event
1	PT1	1	1.705	8072	260	0.15	Eight figure of knot	1	Retained
2	PT1	2	1.680	8657	300	0.18			Retained
3	PT2	3	1.740	6095	350	0.20			Retained
4	PT2	4	1.705	7091	345	0.20			Retained
5	T1	5	1.650	10620	87	0.08	Commercialized		Retained
6	T1	6	1.690	9583	70	0.04			Retained
7	T2	7	1.730	13,047	30	0.02			Retained
8	T2	8	1.670	11,107	40	0.02			Retained

**Table 6:** Test of specimens. Maximum arrest force and elongation in FF = 2.

Specimen	Type	Test	Length with Connectors (m)	Maximum Arrest Force (N)	Lanyard Elongation (mm)	Strain (m/m)	Terminal Type	FF	Event
1	PT1	9	1.696	12,632	354	0.21	Figure-of-eight knot	2	Retained
2	PT1	10	1.685	6687	-	-			Snapped
3	PT2	11	1.712	8521	648	0.38			Retained
4	PT2	12	1.762	9752	440	0.25			Retained
5	T1	13	1.693	16,178	97	0.06	Commercialized		Retained
6	T1	14	1.692	8228	-	-			Snapped
7	T2	15	1.733	5455	-	-			Snapped
8	T2	16	1.674	7941	-	-			Snapped

**Table 7:** Average results of lanyards studied.

Type	FF	Average Length (m)	Average Maximum Arrest Force (kN)	Average Elongation (mm)	Average Strain (m/m)
PT (prototype)	1	1.708	7.48	314	0.183
T (commercialized)	1	1.685	11.09	57	0.040
PT (prototype)	2	1.714	10.30	481	0.280
T (commercialized	2	1.698	16.18	97	0.060
